# Screening high-risk patients and assisting in diagnosing anxiety in primary care: the Patient Health Questionnaire evaluated

**DOI:** 10.1186/1471-244X-13-192

**Published:** 2013-07-17

**Authors:** Anna DT Muntingh, Eric W De Heer, Harm WJ Van Marwijk, Herman J Adèr, Anton JLM Van Balkom, Philip Spinhoven, Christina M Van der Feltz-Cornelis

**Affiliations:** 1 Netherlands Institute of Mental Health and Addiction (Trimbos Institute), PO box 725, Utrecht 3500AS, The Netherlands; 2 Faculty of Social Sciences, Tranzo department, Tilburg University, PO Box 90153, Tilburg 5000 LE, The Netherlands; 3 EMGO Institute for Health and Care Research (EMGO+), PO Box 7057, Amsterdam 1007 MB, The Netherlands; 4 Department of General Practice and Elderly Care Medicine, VU University Medical Centre, Van der Boechorststraat 7, Amsterdam 1081BT, The Netherlands; 5 Johannes van Kessel Advising, Wederkuil 11, Huizen 1273SB, The Netherlands; 6 Department of Psychiatry, VU University Medical Centre and GGZ inGeest, A.J. Ernststraat 1187, Amsterdam 1081 HL, The Netherlands; 7 Institute of Psychology, Leiden University, PO Box 9555, Leiden 2300 RB, The Netherlands; 8 Department of Psychiatry, Leiden University Medical Centre, PO Box 9600, Leiden 2300 RC, The Netherlands; 9 Clinical Centre for Body, Mind and Health, GGZ Breburg, Lage Witsiebaan 4, Tilburg 5042 DA, The Netherlands

**Keywords:** Anxiety disorder, Patient health questionnaire, Primary care, Screening, Case finding, Criterion validity

## Abstract

**Background:**

Questionnaires may help in detecting and diagnosing anxiety disorders in primary care. However, since utility of these questionnaires in target populations is rarely studied, the Patient Health Questionnaire anxiety modules (PHQ) were evaluated for use as: a) a screener in high-risk patients, and/or b) a case finder for general practitioners (GPs) to assist in diagnosing anxiety disorders.

**Methods:**

A cross-sectional analysis was performed in 43 primary care practices in the Netherlands. The added value of the PHQ was assessed in two samples: 1) 170 patients at risk of anxiety disorders (or developing them) according to their electronic medical records (high-risk sample); 2) 141 patients identified as a possible ‘anxiety case’ by a GP (GP-identified sample). All patients completed the PHQ and were interviewed using the Mini International Neuropsychiatric interview to classify DSM-IV anxiety disorders. Psychometric properties were calculated, and a logistic regression analysis was performed to assess the diagnostic value of the PHQ.

**Results:**

Using only the screening questions of the PHQ, the area under the curve was 83% in the high-risk sample. In GP-identified patients the official algorithm showed the best characteristics with an area under the curve of 77%. Positive screening questions significantly increased the odds of an anxiety disorder diagnosis in high-risk patients (odds ratio = 23.4; 95% confidence interval 6.9 to 78.8) as did a positive algorithm in GP-identified patients (odds ratio = 13.9; 95% confidence interval 3.8 to 50.6).

**Conclusions:**

The PHQ screening questions can be used to screen for anxiety disorders in high-risk primary care patients. In GP-identified patients, the benefit of the PHQ is less evident.

## Background

In health care systems in which the general practitioner (GP) acts as the gatekeeper to mental health care, the ability of GPs to accurately detect and diagnose psychiatric disorders is crucial. Anxiety disorders are a major category of the psychiatric disorders encountered in primary care. The ability of GPs to detect anxiety disorders has often been criticised [1] as GPs only detect one third to one half of the patients with an anxiety disorder [2-4]. Although GPs do suspect psychological problems in many of these patients, they do not often classify these problems with a diagnosis of an anxiety disorder [5]. However, the classification of an anxiety disorder diagnosis facilitates the implementation of clinical guidelines, as these include diagnosis-specific treatments [4,6]. Furthermore, the provision of multidisciplinary care may be enhanced by the use of common terms by GPs and mental health professionals [7,8].

To increase the identification and, ultimately, treatment of patients with anxiety disorders in primary care, screening is often considered [9]. Screening is intended to identify individuals at risk of having or developing a disorder. There are several questionnaires available that have been shown to be valid instruments to screen for anxiety disorders in primary care [10-13]. However, screening large populations of patients for mental disorders is generally not considered efficient as this requires a substantial investment and may be of limited effectiveness in increasing recognition and treatment [14]. We clearly need a more efficient approach to identify patients with anxiety disorders who are likely to profit from treatment. It has been proposed to purposefully screen patients who are at high risk of developing a disorder [15]. Another approach that has already been suggested in guidelines for depression [16] and anxiety [17] is to help the GP to distinguish distress symptoms from an anxiety disorder [18] by using a screening instrument (case finding). However, evidence of the value of available screening instruments in screening high-risk groups (selective screening) and in assisting GPs in diagnosing an anxiety disorder is limited. The Patient Health Questionnaire (PHQ) might be suitable for this purpose because this scale was specifically designed for use in primary care [13] and has shown adequate psychometric properties [13,19,20]. The PHQ consists of different modules about common mental health disorders, including a module about panic disorder and one about general anxiety. Earlier studies about the PHQ anxiety module focused on the validity as a screener in a random primary care sample [13], different groups of hospital patients [19-21], the community [22], and in psychosomatic outpatients [23]. A recent study showed that the ability of the PHQ panic module to detect panic disorder in high-risk primary care patients was moderate [24]. However, most GPs will be interested in the presence of any anxiety disorder, after which they may decide to perform extra diagnostic procedures or refer the patient to a mental health professional.

In conclusion, information about the practical value of existing screening instruments for anxiety disorders in primary care is insufficient. We therefore evaluated the PHQ anxiety modules for use as: a) a screener in high-risk patients, and/or b) a case finder for general practitioners to assist in diagnosing anxiety disorders in a pragmatic diagnostic study.

## Method

### Participants

Patients were recruited between November 2008 and March 2010 in 43 primary care practices. Two samples of patients were studied: 1) primary care patients at risk of anxiety disorders (or developing them), identified via their electronic medical record (EMR) (high-risk sample); 2) patients identified as a possible ‘anxiety case’ by their GP (GP-identified sample). Except for their entrance into the study (selection from the EMR or identification by their GP), patients followed the same procedure. This study was performed in primary care practices that participated in a cluster randomised controlled trial (RCT) focusing on the treatment of panic disorder and generalised anxiety disorder in primary care [25].

### Exclusion criteria

We excluded patients who were suicidal, patients who suffered from dementia or other severe cognitive disorders, psychotic disorder, bipolar disorder and dependence on drugs or alcohol. Other exclusion criteria were insufficient knowledge of the Dutch language to complete the questionnaire and receiving regular psychological treatment.

### Selection of the high-risk sample

To select patients who were at risk of anxiety disorders (or developing them), electronic medical records (EMRs) were searched. Of the 43 practices participating in the RCT, 24 practices agreed to the screening procedure and made use of an electronic system suitable for selecting patients from the EMR. Patients were selected from the EMR if they were over 18 years of age and had visited their general practitioner in the previous three months with symptoms that were considered to indicate a high risk of anxiety disorders. Such symptoms were fatigue, headache, dizziness, weakness, muscle- and joint pain, stomach ache, chest pain, hyperventilation, anxiety or depression (or symptoms thereof), or social problems (such as loneliness or marital problems). These symptoms have been identified as risk factors for having or developing anxiety disorders [26-28].

### Selection of the GP-identified sample

GPs were asked to identify patients with an anxiety disorder (specifically panic disorder and generalised anxiety disorder). Because the GPs participated in an RCT, they received instructions to refer patients to the study if they judged them to have an anxiety disorder. Instructions were based on the national guidelines for GPs, which are available to all GPs in the Netherlands. GPs (N = 37) who were allocated to the intervention group of the RCT received these instructions during a meeting in which the RCT was explained. GPs in the control group (N = 26) of the RCT received an educational folder.

### Procedure

Patients who were selected from the EMR (high-risk sample) and patients who were identified by their GP (GP-identified sample) received a letter informing them about the RCT, together with an informed consent form and the Patient Health Questionnaire (PHQ) anxiety module. The patients were asked to return the PHQ and the informed consent form directly to the researcher. They were not informed about the allocation of their GP in the RCT. Patients who gave informed consent were contacted by telephone by a research assistant to perform a diagnostic interview.

### Measures

#### Screener

The PHQ anxiety modules [13] consist of 22 questions concerning anxiety symptoms experienced during the previous four weeks. The first 15 questions screen for a panic disorder, starting with four questions about the presence of panic attacks and anxiety (first screening question: “In the last 4 weeks, have you had an anxiety attack – suddenly feeling fear or panic?”) and subsequently asking about symptoms of panic attacks, such as “Were you short of breath?”. The second part of the PHQ consists of seven characteristics of generalised anxiety, starting with a screening question “Feeling nervous, anxious, on edge, or worrying a lot about different things”, followed by symptoms of generalised anxiety. The respondents are asked to indicate how often they were bothered by these problems (“not at all”, “several days” or “more than half the days”). Good overall accuracy has been reported for both subscales [13,19,23] and a good test-retest reliability (Kappa 0.84) of the PHQ panic module has been reported in chronic Hepatitis C patients [29]. Both modules are scored using an algorithm which leads to a conclusion about the likely presence (positive algorithm) or absence (negative algorithm) of the anxiety disorder. If the first four questions of the panic module were answered affirmatively, and at least four symptoms of panic attacks were present, this was counted as a positive algorithm [13]. If the first question of the generalised anxiety module was answered with “more than half the days” and at least three symptoms of generalised anxiety were present for more than half the days, this was also counted as a positive algorithm [13]. If a negative algorithm was scored on both modules, this was counted as a negative algorithm. We also tested the diagnostic validity of the two screening questions, since the use of this simplified algorithm may be more suitable for high-risk groups [24] and is more useful in busy general practices. An affirmative answer to the first screening question of the panic module or the answer “more than half the days” to the screening question of the generalised anxiety module was considered as positive screening questions.

#### Reference standard

The Mini International Neuropsychiatric interview (MINI-PLUS) was used as a reference standard and to assess exclusion criteria. MINI-PLUS is a short structured diagnostic interview that is used to determine the most common DSM-IV [30] and ICD-10 [31] psychiatric disorders [32] (Dutch version [33]). The following anxiety disorders were classified: panic disorder (PD) with or without agoraphobia, generalised anxiety disorder (GAD), social phobia, simple phobia, obsessive compulsive disorder, post-traumatic stress disorder and agoraphobia (without panic disorder). Depressive disorders, suicidal ideation, psychotic disorders and substance use disorders were also assessed using the MINI-PLUS. The interviewers who conducted the MINI interviews by telephone had a medical or psychological background, with degrees varying from bachelor to master. They received training in how to carry out the MINI interview and were supervised by a psychologist and a psychiatrist. At least two interviews carried out by each interviewer were audio-taped and evaluated by the psychologist. The interviewers had the opportunity to verify their diagnosis with a study psychiatrist. All interviewers and the study psychiatrist were blinded for the PHQ score to prevent confirmation bias.

### Recruitment

#### High-risk sample

**Figure 1:**
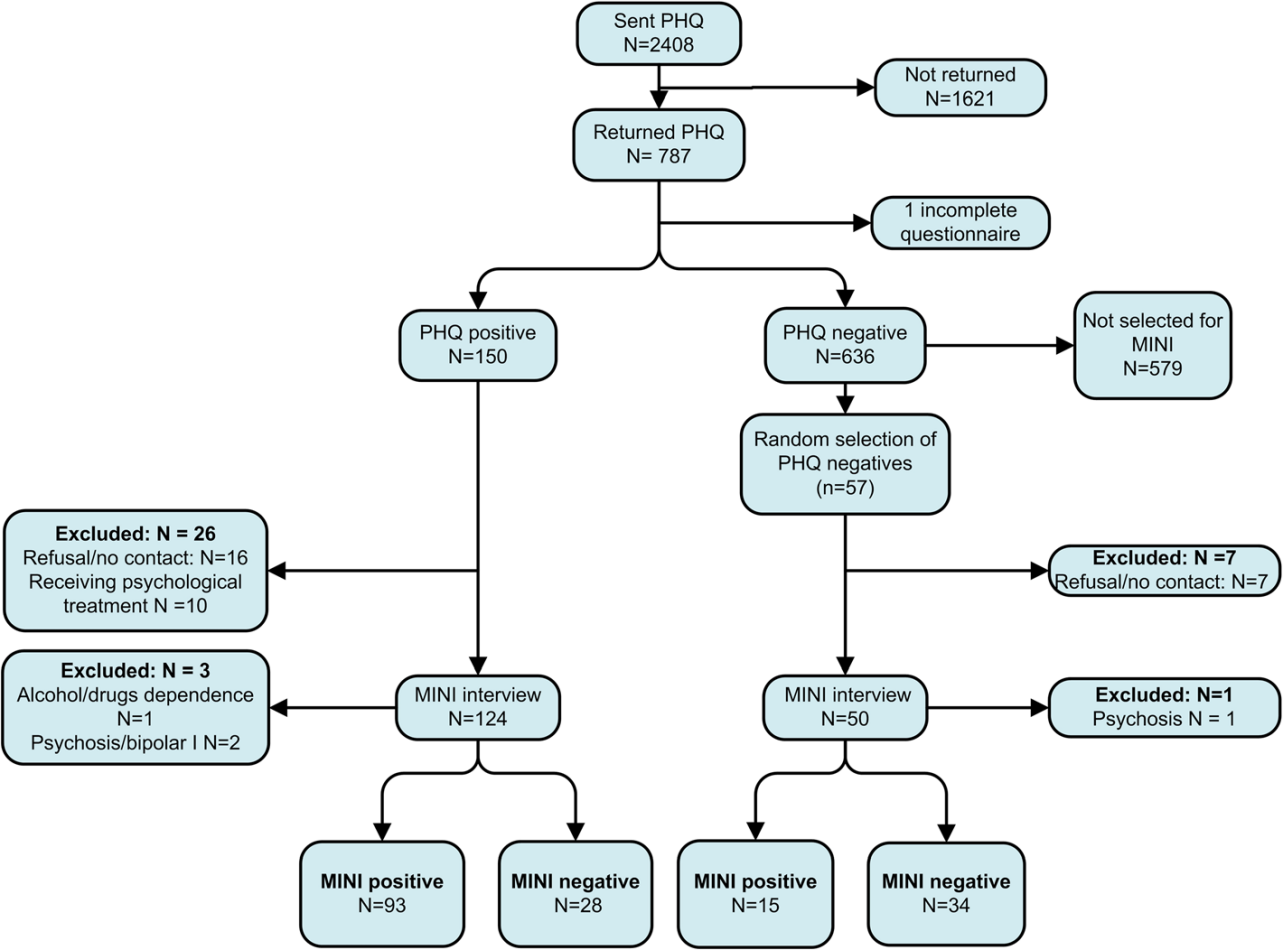
**Flowchart of participants at risk of an anxiety disorder (or of developing one) according to their electronic medical record (high**-**risk sample).**

The PHQ was sent to 2,408 patients who were at risk of developing an anxiety disorder according to the information in their EMR. A total of 786 (32.6%) patients completed and returned the questionnaire (Figure 1). The proportion of females did not differ significantly between responders and non-responders (70% versus 69%, p > 0.05) but the responders were slightly older than the non-responders (51.9 versus 48.3 years, p < .05). All patients with a positive algorithm (N = 150), and a random selection of 57 patients with a negative algorithm were invited for a MINI interview. After the exclusion of patients who met exclusion criteria (N = 13, 8.7% positive algorithm, N = 1, 2% negative algorithm), and due to non-response (N = 16, 10.7% positive algorithm, N = 7, 10.5% negative algorithm), 121 participants with a positive algorithm and 49 participants with a negative algorithm had a MINI interview (n = 170). Figure 1 presents a flowchart of the high-risk sample.

#### GP-identified sample

GPs of 37 practices selected 207 patients for the study. All patients who gave informed consent and who did not meet the exclusion criteria (N = 164, 79.2%) were invited for a MINI interview, irrespective of their PHQ score, and eventually 141 patients were interviewed.

**Figure 2:**
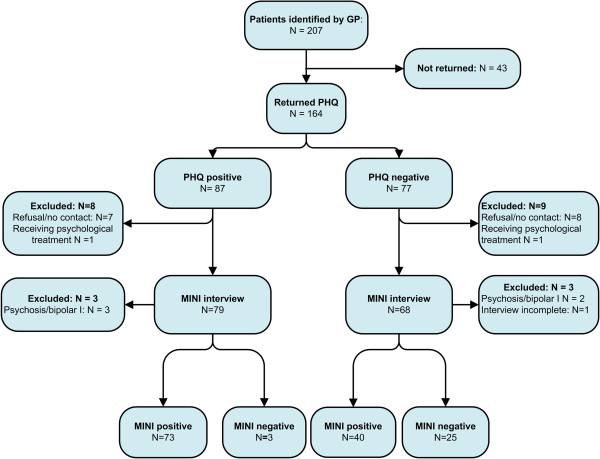
**Flowchart of patients identified as a case by GPs (GP**-**identified sample).**

As we found no significant difference in the diagnostic accuracy of GPs randomised to the intervention or control group of the RCT (data not shown), we analysed the total group of patients identified by their GP. Figure 2 presents a flowchart of patients identified by their GP.

#### Data analysis

In the high-risk sample, we had to correct for the fact that we included a random sample of patients with a negative algorithm, while we did include all patients with a positive algorithm. Such a selection procedure creates an imbalance that influences the prevalence and thus the test characteristics. Weights were used to transform the sample back to the original distribution of positive and negative algorithms [34]. A positive algorithm patient received a weight of 0.27 (150/121*170/786) and a negative algorithm patient received a weight of 2.81 (636/49*170/786). A similar procedure was followed for the analysis in the high-risk sample using the two screening questions. All psychometric analyses concerning the high-risk group were performed on the weighted sample. The following indicators of criterion validity were calculated: positive predictive value, negative predictive value, sensitivity, specificity, overall accuracy and receiver operating characteristics (area under the curve, AUC) [35]. The MINI classification functioned as reference standard for the diagnosis of an anxiety disorder. A multilevel logistic regression analysis was performed to determine whether a positive algorithm or positive screening questions increased the odds of a MINI anxiety disorder classification. SPSS version 15.0 was used for the majority of the statistical analyses; MLwiN V2 2.21 [36] was used for the multilevel logistic regression analysis.

## Results

### Participants

In the weighted high-risk sample (N = 170) the mean age was 54.6 (SD 13.2), the percentage of females was 74% and the prevalence of any anxiety disorder was 39%. In the GP-identified sample (N = 141), the mean age was 47.5 (SD 16.4), the percentage of females was 71% and the prevalence of any anxiety disorder was 80% (Table 1).

**Table 1:** **Characteristics of the high**-**risk sample** (**weighted**) **and the GP**-**identified sample**

	**1) High**-**risk sample (N** **=** **170)**	**2) GP**-**identified sample (N** **=** **141)**
**N (%)**		
**Mean age (range)**	54.6 (19–82)	47.52 (18–83)
**Female**	124 (74.4%)	100 (70.9%)
**Male**	43 (25.2%)	41 (29.1%)
Anxiety disorder diagnosis*	67 (39.4%)	113 (80.1%)
***Panic disorder***	*23* (*13*.*5*%)	*78* (*55*.*3*%)
***Generalised anxiety disorder***	*23* (*13*.*5*%)	*52* (*36*.*9*%)
***Social phobia***	*17* (*10*.*1*%)	*29* (*20*.*6*%)
***Simple phobia***	*11* (*6*.*7*%)	*20* (*14*.*2*%)
***Obsessive compulsive disorder***	*2* (*0*.*9*%)	*14* (*9*.*9*%)
***Post***-***traumatic stress disorder***	*1* (*0*.*3*%)	*5* (*3*.*5*%)
***Agoraphobia***	*19* (*11*.*0*%)	*4* (*2*.*8*%)
***Major depressive disorder***	*11* (*6*.*5*%)	*19* (*13*.*5*%)

*Percentages do not total to 100% since many patients had more than one anxiety disorder.

### Test results

The results are summarised in Table 2. For the high-risk sample, the two screening questions of the PHQ showed the best test characteristics, due to an increase in sensitivity. The positive predictive value (PPV) was 76%, the negative predictive value (NPV) was 88% and the overall performance of the PHQ as expressed by the area under the curve was 83%, which may be interpreted as moderate to high [35]. In GP-identified patients, the official algorithm performed best, with a PPV of 96%, an NPV of 38% and the area under the curve was moderate (77%).

**Table 2:** **Psychometric properties of the PHQ disorder in a high**-**risk sample** (**weighted**) **and a GP**-**identified sample**

	**High**-**risk sample (N** **=** **170)**								**GP**-**identified sample (N** **=** **141)**							
	**Official algorithm**^**a**^				**Screening questions**^**b**^				**Official algorithm**^**a**^				**Screening questions**^**b**^			
**Cross**-**tabulation**	a	b	c	d	a	b	c	d	a	b	c	d	a	b	c	d
	25	8	42	95	49	15	12	93	73	3	40	25	106	21	7	7
**Prevalence**	0.39 (67/170)				0.36 (61/170)				0.80 (113/141)				0.80 (113/141)			
**Positive predictive value**	0.77 (25/32)				0.76 (49/64)				0.96 (73/76)				0.84 (106/127)			
	[0.71–0.83]				[0.70–0.83]				[0.93–0.99]				[0.77–0.90]			
**Negative predictive value**	0.69 (95/138)				0.88 (93/105)				0.38 (25/65)				0.50 (7/14)			
	[0.62–0.76]				[0.83–0.93]				[0.30–0.46]				[0.42–0.58]			
**Positive likelihood ratio**	5.1 [4.41–5.79]				5.71 [4.93–6.49]				6.03 [5.12–6.94]				1.25 [1.16–1.35]			
**Negative likelihood ratio**	0.68 [0.61–0.75]				0.23 [0.17–0.30]				0.40 [0.32–0.48]				0.25 [0.18–0.32]			
**Sensitivity**	0.37 (25/67)				0.80 (49/61)				0.65 (73/113)				0.94 (106/113)			
	[0.30–0.44]				[0.74–0.86]				[0.57–0.72]				[0.90–0.98]			
**Specificity**	0.93 (95/103)				0.86 (93/108)				0.89 (25/28)				0.25 (7/28)			
	[0.89–0.97]				[0.81–0.91]				[0.84–0.94]				[0.18–0.32]			
**Overall accuracy**	0.71 (95 + 25/170)				0.84 (93 + 49/170)				0.70 (73 + 25/141)				0.80 (106 + 7/141)			
	[0.64–0.78]				[0.78–0.89]				[0.62–0.77]				[0.74–0.87]			
**Area under the curve**	0.65*				0.83*				0.77 [0.68–0.86]				0.60 [0.47–0.72]			
	[0.64–0.66]				[0.82–0.84]											

a: true positives, b: false positives, c: false negatives, d: true negatives, ^a^ Official algorithm panic disorder: All the first four questions were answered with “yes” and four symptoms related to panic attacks are present. Official algorithm general anxiety: The first question was answered with “more than half the days” and three symptoms related to general anxiety were present more than half the days. ^b^ Screening question for panic disorder (“In the last 4 weeks, have you had an anxiety attack - suddenly feeling fear or panic?”) was answered with yes AND/OR screening question for general anxiety (“Feeling nervous, anxious, on edge, or worrying a lot about different things”) was answered with “more than half the days”. *Because weighting in the receiver operating characteristics (ROC) analysis is only possible with integer values, the weights were multiplied by 100).

### Diagnostic value of the PHQ

In the high-risk sample, a positive algorithm significantly increased the odds of a MINI anxiety disorder classification (odds ratio 6.7; 95% confidence interval (CI) 3.0-14.6) and positive screening questions even more so (odds ratio 23.4; 95% CI 6.9-78.8). In the GP-identified patients, a positive algorithm significantly increased the odds of a MINI classification (odds ratio 13.8; 95% CI 3.8-50.6) as did positive screening questions, although to a lesser extent (odds ratio 7.3; 95% CI 2.1 – 25.7).

## Discussion

### Summary of main findings

The results imply that the PHQ screening questions may be used to screen for anxiety disorders in high-risk groups. The official algorithm may be useful as an adjunct to the clinical diagnosis made by the GP. In the high-risk sample, the performance of the PHQ using the official algorithm was moderate, but the two screening questions of the PHQ showed good test characteristics. In the GP-identified sample, a positive algorithm adequately predicted the presence of an anxiety disorder but the ability of the PHQ to filter out non-cases was inadequate in these patients.

### Strengths and limitations of the study

A strength of this study is its focus on the practical purpose of screening and case finding.

A limitation is that patients were recruited in primary care practices that participated in an RCT. However, exclusion criteria from the RCT do not seem to have had a significant influence on the results, as only 29 out of 951 patients (3%) who returned the PHQ were eventually excluded based on the exclusion criteria of the RCT. Our population was clearly broader than the population of the RCT; eventually, 180 (58%) out of 311 patients participated in the RCT. Another limitation was the low response rate in the high-risk sample. Patients who experienced anxiety problems may have been more likely to return the questionnaire and therefore the prevalence of anxiety disorders in our study may be overestimated. However, a similar response pattern may also be expected if a screening procedure were to be conducted in daily practice and therefore is also consistent with our aim of assessing the practical value of the PHQ.

Because GPs in our study may have had an increased interest in anxiety disorders and received instructions on the recognition of anxiety disorders, they may have had a greater ability to recognise anxiety disorders than seen in the total population of GPs. However, instructions were minimal and based on the national guidelines for GPs (which are available to all GPs in the Netherlands) and we consider it unlikely that this has significantly influenced the skills of the GPs.

### Comparison with existing studies

This study confirms that the characteristics of patients influence test characteristics [37]. It is therefore relevant to test the value of a questionnaire in different populations and to relate findings to those of similar studies. The performance of the PHQ in our high-risk sample is consistent with the performance of the panic module in a previous study with a high-risk sample consisting of frequent attenders, patients with medically unexplained symptoms, and patients with mental health problems in primary care [24]. The authors also found that using only the screening questions improved the performance of the PHQ substantially and concluded that the PHQ was of moderate value for screening in high-risk groups. Other studies have found a better performance of the PHQ [13,19,20]. This may be due to the characteristics of our study population (patients at risk of anxiety disorders), with our study design (cross-sectional analysis within an RCT), or with the characteristics of the instrument itself. Other screening instruments are available for anxiety disorders that have been tested in primary care and that showed adequate characteristics, such as the Generalised Anxiety Disorder-7 item scale (GAD-7) [38], the Hospital Anxiety and Depression Scale [39], and the Four Dimensional Symptom Questionnaire (4DSQ) [40]. The GAD-7, developed by the same research group that produced the PHQ, may be more accurate than the PHQ in detecting anxiety disorders [41]. This may be in part due to the fact that the GAD-7 contains three items that differ from the PHQ items and, moreover, because it yields a continuous score that allows a selection of an optimal cutpoint. While the original study showed good AUCs for the GAD-7 in detecting anxiety disorders other than GAD, differing results from a recent study [37] suggest that the performance of the GAD-7 for anxiety disorders other than GAD needs further research. As there is no direct comparison available between the GAD-7 and the PHQ, there is no decisive information yet on which scale performs best. The HADS has also shown adequate characteristics in primary care patients [11,12]. However, just as with the GAD-7, limited information is available about the practical value of the HADS in high-risk primary care patients or GP-identified patients. One study comparing the HADS and the 4DSQ in GP-identified patients has shown areas under the curve [10] similar to the area under the curve of 77% found in our study.

### Implications for future research and practice

The high prevalence of anxiety disorders (39%) in the high-risk sample suggests that selecting patients from the EMR on the basis of psychological symptoms, social problems or physical symptoms related to anxiety disorders might be a successful method for selective screening in primary care. This may be especially relevant for patients presenting with physical symptoms as it is difficult for GPs to recognise anxiety disorders in these patients [42]. The finding that the use of the two screening questions of the PHQ resulted in the best performance is positive, as this makes the screening procedure short. However, when implementing a selective screening procedure, it needs to be followed by a structured approach of further clinical diagnostic procedures and evidence-based treatment, as recommended in clinical guidelines [6]. Therefore, low-intensity interventions need to be available in primary care to be able to treat a large number of patients [43]. Only then will selective screening be an effective way of improving management of anxiety disorders. With regard to GPs who referred patients to this study, it is noteworthy that they did correctly suspect the presence of an anxiety disorder in 80% of the cases. However, the number of patients that GPs identified varied widely (from 0 to 17). Efforts to improve detection of anxiety disorders may thus be aimed at GPs with a low recognition rate of anxiety disorders. Furthermore, it may be worthwhile to prompt GPs to investigate the presence of an anxiety disorder with the PHQ in patients with less obvious anxiety symptoms. We recommend that future diagnostic studies should pay attention to the practical purposes of screening instruments, to help informing primary care on the best way to use these instruments. Additional research is also necessary to investigate the most effective method for screening for anxiety disorders in terms of higher treatment rates [44,45] and improved patient outcomes [14].

## Conclusions

The results of this study show that the two screening questions of the PHQ form a suitable instrument for screening for anxiety disorders in high-risk primary care patients. Although GPs may use the official algorithm of the PHQ in adjunct to their clinical diagnosis, they are not advised to use the PHQ for the purpose of ruling out the presence of an anxiety disorder. Following this study, research should focus on the effectiveness of selective screening for anxiety disorders in primary care and on strategies to improve the recognition of anxiety disorders by GPs with a low recognition rate.

### Ethics approval

VU University Medical Ethics Committee.

## Abbreviations

4DSQ: Four dimensional symptom questionnaire; AUC: Area under the curve; CI: Confidence interval; DSM-IV: Diagnostic and statistical manual of mental disorders; EMR: Electronic medical record; GAD-7: Generalised anxiety Disorder-7 item scale; GP: General practitioner; HADS: Hospital anxiety and depression scale; ICD-10: International statistical classification of diseases; LR+: Positive likelihood ratio; LR-: Negative likelihood ratio; M: Mean; MINI-PLUS: MINI International Neuropsychiatric Interview; NPV: Negative predictive value; OR: Odds ratio; PHQ: Patient health questionnaire; PPV: Positive predictive value; RCT: Randomised controlled trial; ROC: Receiver operating characteristics; SD: Standard deviation.

## Competing interests

The authors declare that they have no competing interests.

## Authors’ contributions

AM participated in the design of the study, data collection, data analysis, and wrote this article. EdH participated in data collection, data analysis and in writing the article. HvM and PhS advised on data analysis and co-authored the article. HA advised on the design and data analysis, performed statistical analyses and co-authored the article. TvB advised on and co-authored the article. CFC participated in the design of the study, supervised data collection and analysis and co-authored the article. All authors read and approved the final manuscript.

## Pre-publication history

The pre-publication history for this paper can be accessed here:

http://www.biomedcentral.com/1471-244X/13/192/prepub
